# Impact of Spatial LAI Heterogeneity on Estimate of Directional Gap Fraction from SPOT-Satellite Data

**DOI:** 10.3390/s8063767

**Published:** 2008-06-06

**Authors:** Lingling Ma, Chuanrong Li, Bohui Tang, Lingli Tang, Yuyin Bi, Beiyan Zhou, Zhao-Liang Li

**Affiliations:** 1 Institute of Remote Sensing Application, Chinese Academy of Sciences, Beijing, 100101, China; E-mail: llma@aoe.ac.cn; 2 Graduate University of Chinese Academy of Sciences, Beijing, 100049, China; 3 Academy of opto-electronics, Chinese Academy of Sciences, Beijing, 100080, China; E-mails: crli@aoe.ac.cn; lltang@aoe.ac.cn; 4 Institute of Geographic Sciences and Natural Resources Research, Chinese Academy of Sciences, Beijing, 100101, China; E-mails: tangbh@igsnrr.ac.cn; lizl@igsnrr.ac.cn; 5 Institute of Agricultural Resources and Regional Planning, Chinese Academy of Agricultural Sciences, Beijing, 100081, China; E-mail: biyuyun@sina.com; 6 SinoMaps Press, Beijing, 100054, China; E-mail: zhoubeiyan1999@126.com

**Keywords:** directional gap probability, scaling bias, leaf area index, clumping index

## Abstract

Directional gap probability or gap fraction is a basic parameter in the optical remote sensing modeling. Although some approaches have been proposed to estimate this gap probability from remotely sensed measurements, few efforts have been made to investigate the scaling effects of this parameter. This paper analyzes the scaling effect through aggregating the high-resolution directional gap probability (pixel size of 20 meters) estimated from leaf area index (LAI) images of VALERI database by means of Beer's law and introduces an extension of clumping index, Ĉ, to compensate the scaling bias. The results show that the scaling effect depends on both the surface heterogeneity and the nonlinearity degree of the retrieved function. Analytical expressions for the scaling bias of gap probability and Ĉ are established in function of the variance of LAI and the mean value of LAI in a coarse pixel. With the VALERI dataset, the study in this paper shows that relative scaling bias of gap probability increases with decreasing spatial resolution for most of land cover types. Large relative biases are found for most of crops sites and a mixed forest site due to their relative large variance of LAI, while very small biases occur over grassland and shrubs sites. As for Ĉ, it varies slowly in the pure forest, grassland and shrubs sites, while more significantly in crops and mixed forest.

## 1. Introduction

Directional gap probability or gap fraction is defined originally as the probability of a beam transferring at a given incident zenith angle through the vegetative canopy without any interception. As a key variable describing canopy structure and biomass spatial distribution, it is used to simplify the 3- D light interception problem to a 1-D problem (Pinty et al., 2004), and has been employed to estimate surface component temperatures from multi-spectral and multi-angular measurements (Francois and Ottle, 1997; Francois, 2002, Li et al., 2001; Menenti et al, 2008). Though gap probability can be estimated in situ from optical instrument data such as hemispherical photographs (Leblanc et al., 2005) and usually used to derive leaf area index (LAI) at local scale in field (Jonckheere et al., 2004; Weiss et al., 2004), the field measurements cannot meet the practical demands at large scale. An attractive and unique way to map and monitor LAI and directional gap probability at large scale is to use the space observation from satellite data in the visible and near-infrared bands. Nowadays LAI is widely estimated directly from satellite measurements using different methods (Myneni et al., 1997; Weiss and Baret, 1999; Chen et al., 2002; Fernandes et al., 2003) and the directional gap probability P is estimated from the spatially retrieved LAI by means of the following relationship (Norman, 1995; Menenti et al., 2001),

(1) $$P(\theta ,\text{LAI})=e^{-\text{GLAI}/\cos(\theta )}$$

where *θ* is the zenith angle of incident beam, G is the projection of leaf area in perpendicular to incident beam and is related to the leaf angle distribution (Wang et al., 2007). With this relationship, directional gap probability can be estimated through vegetation structure information including LAI, leaf angle distribution.

Through observation and studies in different scales including foliage (Rochdi and ad M. Chelle, 2006), shoot (Smolander and Stenberg, 2003), canopy (Kotz et al., 2004) and landscape (Garrigues et al., 2006a) by remote sensing, ecological and agricultural community, scientists have realized spatial heterogeneity is universal. Besides the spatial heterogeneity of the land surface, non-linearity of the transfer function is another source of uncertainties in the estimation of land surface variables/parameters from remotely sensed data. (Hall et al., 1992; Friedl et al., 1995; Pelgrum, 2000; Garrigues, 2006b). We can notice that the directional gap probability P estimated from equation 1 is highly non-linear with respect to LAI, which will inevitably induced scaling bias when applied to a coarse pixel. Consequently it is necessary to analyze the scaling effect of the directional gap probability at different scales, and to improve the retrieval accuracy of directional gap probability, and subsequently to improve the accuracy of land surface component temperatures retrieved from multi- spectral and multi-angular satellite data. However, up to now, there are no many efforts in literature devoted to study the scaling effect of the directional gap probability.

This study focuses on the analysis of the scaling effect on the directional gap probability by means of a simple scaling-up scheme and LAI derived from high resolution spatial data. The second section provides the theoretical framework to estimate the scaling effect of directional gap probability raised by two different aggregation schemes from local scale to larger scale. In the third section, we present the different types of remotely sensed LAI images obtained from VALERI database (Validation of Land European Remote sensing Instruments). In section 4, the scaling effect associated with the non- linear relationship between LAI and gap probability is quantified over several types of landscape. Conclusion is given in section 5.

## 2. Theoretical framework

### 2.1. Up-scaling of directional gap probability

**Figure 1.**
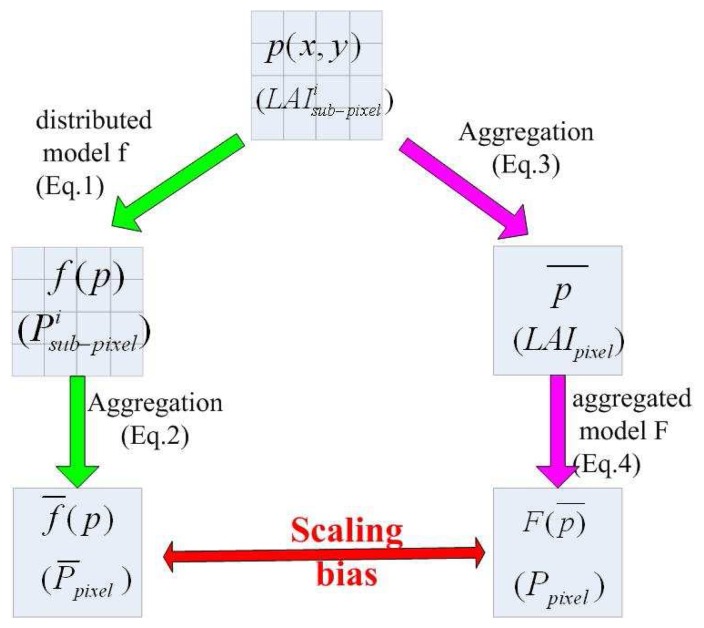
General schemes of two aggregation schemes.

There are two different schemes generally used to aggregate the parameters/variables from the local scale to regional or global scale (Pelgrum, 2000), which are depicted in Figure 1 and described roughly below:

1) The aggregation of the results which are derived from a distributed model f using distributed input variables. Spatially distributed variables *p*(*x*, *y*) (here $LAI_{\text{sub}-\text{pixel}}^{i}$) are input to a distributed model f (here Eq. 1), results of the distributed model f are denoted as *f* (*p*) (here $P_{\text{sub}-\text{pixel}}^{i}(\theta )$), then the aggregative result *f̅*(*p*) (here *P̅_pixel_*(*θ*)) on a larger scale are deduced (Eq. 2) from distributed results;(see left flow chart of Figure 1)
2) The aggregation of input variables before use in an aggregative model F (here Eq. 3), thereby producing an aggregative result. Spatially distributed input data *p*(*x*, *y*) (here $LAI_{\text{sub}-\text{pixel}}^{i}$) are first averaged to *p̅* (here *LAI_pixel_*) from local scale to a larger scale, then *p̅* is input to aggregative model F (Eq. 4), produces aggregative result *F*(*p̅*) (here *P_pixel_*(*θ*)). (see right flowchart of Figure 1)

As it concerned to gap probability, supposing that the pixel whose area is S is composed by N homogeneous sub-pixels, each sub-pixel i has an area of s_i_ $S=\overset{N}{\underset{i=1}{\text{∑}}}s_{i}$, the directional gap probability for a given direction (i.e. zenith θ) is computed using the first aggregation scheme (see left flowchart of figure 1) with,

(2) $${\bar{P}}_{\text{pixel}}(\theta )=\frac{\sum_{i=1}^{N}s_{i}P_{\text{sub}-\text{pixel}}^{i}(\theta )}{S}$$

where $P_{\text{sub}-\text{pixel}}^{i}$ is the directional gap probability for sub-pixel i, which can be estimated from Eq. 1.

The directional gap probability can also be aggregated following the second aggregation scheme (see right flowchart of figure 1) by

(3) $${\text{LAI}}_{\text{pixel}}=\frac{\sum_{i=1}^{N}s_{i}LAI_{\text{sub}-\text{pixel}}^{i}}{S},$$

Then computing the directional gap probability with help of the same formula as Eq. 1 by

(4) $$P_{\text{pixel}}(\theta )=e^{-{\text{GLAI}}_{\text{pixel}}/\cos(\theta )}$$

### 2.2. Scaling bias of directional gap probability

Since the distributed model related LAI to P is nonlinear (see Eq. 1) and the input LAI data at coarse pixel is heterogeneous, there exists a difference between *p̅_pixel_* and *P_pixel_*. This difference comes from the different aggregations. To assess the scaling effect of the directional gap probability, inserting Eq. 1 into Eq. 2 and neglecting the third and higher order terms of the Taylor series expansion, one gets:

(5) $${\bar{P}}_{\text{pixel}}(\theta )-P_{\text{pixel}}(\theta )=P_{\text{pixel}}(\theta )\frac{G^{2}}{2\cos^{2}(\theta )}\sigma_{\text{LAI}}^{2}$$

with *σ_LAI_* is the standard deviation of LAI inside the coarse pixel, i.e. $\sigma_{\text{LAI}}^{2}=\frac{\sum_{i=1}^{N}s_{i}{\left({\text{LAI}}_{i}-{\text{LAI}}_{\text{pixel}}\right)}^{2}}{S}$

The relative scaling bias (RE) is therefore obtained

(6) $$RE=\frac{{\bar{P}}_{\text{pixel}}(\theta )-P_{\text{pixel}}(\theta )}{P_{\text{pixel}}(\theta )}=\frac{G^{2}}{2\cos^{2}(\theta )}\sigma_{\text{LAI}}^{2}$$

From Eq. 6, we notice that the relative scaling bias is only dependent on the G, *θ* and the spatial heterogeneity of LAI within a coarse pixel, but independent on the LAI value itself.

### 2.3. Redefinition of clumping index

In order to take into account the scaling effects of spatial heterogeneity of LAI on estimate of the directional gap fraction and to make the estimation of the directional gap fraction independent on the observation scale and the aggregation schemes used, a parameter Ĉ is introduced in Eq. 4 so that

(7) $$\exp(-G{\hat{C}}_{\text{pixel}}{\text{LAI}}_{\text{pixel}}/\cos(\theta ))={\bar{P}}_{\text{pixel}}$$

Following the same development made by Wang and Li (2008), combining Eqs 4, 5 and 7, one gets:

(8) $${\hat{C}}_{\text{pixel}}=1-\frac{\cos(\theta )}{{\text{GLAI}}_{\text{pixel}}}\ln\left(1+\frac{G^{2}}{2\cos^{2}(\theta )}\sigma_{\text{LAI}}^{2}\right)$$

As shown by this equation, the parameter Ĉ is directly proportional to the mean LAI and inversely proportional to the spatial heterogeneity of LAI ( $\sigma_{\text{LAI}}^{2}$) for given G function and direction.

It should be noted that the parameter Ĉ introduced in Eq.7 compensate not only the scaling bias in the estimation of the gap probability, but also has the similar meaning as the so-called leaf dispersion parameter or clumping index (Ω). Traditionally, clumping index is generally used to quantify the heterogeneity of the foliage distribution based on Beer-Lambert's law considering a non-random distribution of foliage in a forest canopy, as vegetation foliage is more often grouped together than regularly spaced relative to the random distribution case (Chen, 1996), and vegetative canopies have different levels of foliage organizations, which contribute to non-random distribution (Chen, 1999). For Ω= 1, canopy elements are randomly distributed. In clumped canopies, Ω is always less than unity. The smaller the value of Ω, the more the canopy is clumped.

Foliage clumping affects the gap probability for the same LAI by delaying the occurrence of the saturation in reflectance as LAI increases. There have been some studies mostly concentrated on the estimation of clumping index with multi-angular data. Walter et al. (2003) has conducted an experiment involving hemispherical photographs of simulated and real forest canopies to determine clumping index. Leblanc et al. (2005) and Chen et al. (2005) mapped the foliage clumping index over Canada and at the global scale based on the simulated NDHD-clumping index relationships for different cover types. But the capability of clumping index for representing spatial heterogeneity and eliminating scaling bias is rarely concerned.

## 3. Description of the data

The data used here are part of the VALERI database which provides high spatial resolution (20 m) SPOT-HRV scenes for several landscapes sampled (including crops, forest, grassland and shrubs) around world (Baret et al., 2005). This wide coverage of landscape makes the conclusion of this study more general. Each site has an enough sampling size (about 3km by 3km). Detailed information about each site (including land cover type, location and the date of measurement) is given in table 1. More details on the data set and methodology concerned for leaf area index retrieval is referred to Baret et al. (2005) and the VALERI web site (www.avignon.inra.fr/valeri).

## 4. Results and Discussion

### 4.1. Simulation of relative scaling bias of gap probability

In this study, we adopt a simple formula proposed by Fuchs et al. (1984) to compute the projection value of leaf area in perpendicular to incident beam with mean leaf angle,

(9) $$G=\cos({\bar{\theta }}_{L})$$

where *θ̅_L_* is the mean of leaf inclination angle.

Inserting Eq. 9 into Eq. 6, we get relative scaling bias of gap probability,

(10) $$RE=\frac{\cos^{2}({\bar{\theta }}_{L})}{2\cos^{2}(\theta )}\sigma_{\text{LAI}}^{2}.$$

**Figure 2.**
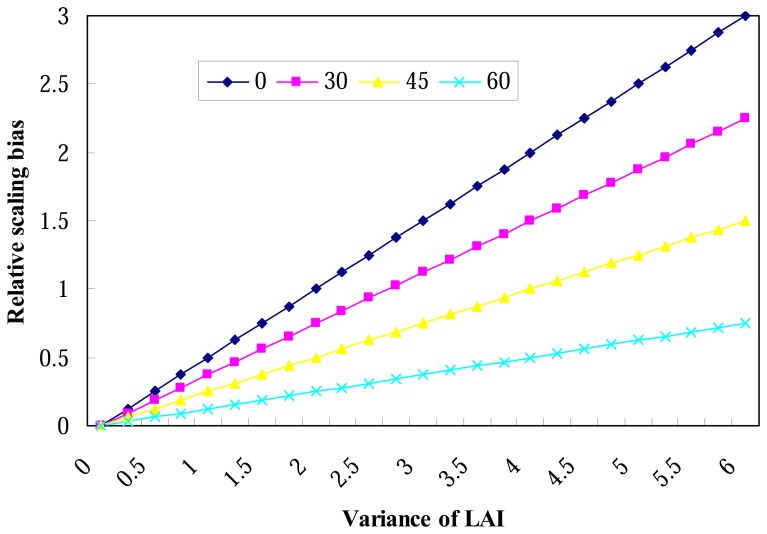
Relative scaling bias of gap probability versus the variance of LAI for different mean of leaf inclination angles *θ̅_L_* (0, 30, 45 and 60 degree) and view zenith angle*θ*= 0.

Figure 2 displays the results of RE conducted using Eq. 10 for θ=0 and different G functions through different mean of leaf inclination angles *θ̅_L_* given in Eq. 9.

As shown in Figure 2, the relative scaling bias of gap probability is linearly related to the variation of LAI inside the coarse pixel for a given mean of leave inclination angle *θ̅_L_*. As predicted by Eq. 10, the slope of this linearity is equal to $\frac{\cos^{2}(\bar{\theta_{L}})}{2\cos^{2}(\theta )}$, and for a given variance of LAI, the larger leaf inclination angle is, the smaller relative error of directional gap probability is. On the other hand, we can conclude that the relative scaling bias varies seasonally since it has relationship with the variance of LAI which is a seasonal variable.

### 4.2. Spatial scaling bias of gap probability obtained from the VALERI dataset

In order to see the magnitude of the spatial scaling bias of directional gap probability with real scenarios, the VALERI dataset is used in this study. Three assumptions are made in the following calculations:

1) Beer's law used to retrieve gap probability from LAI (Eq. 1) is assumed without any scaling bias at 20 m spatial resolution, because no satellite data are available to us at the spatial resolution finer than 20m.
2) Incident beam is assumed to be vertical, i.e. cos(*θ*) = 1
3) A spherical leaf angle distribution is assumed, i.e. G=0.5, which is a reasonable assumption for many conifer shoots and closed, broad-leaved canopies (Walter, 2003).

**Figure 3.**
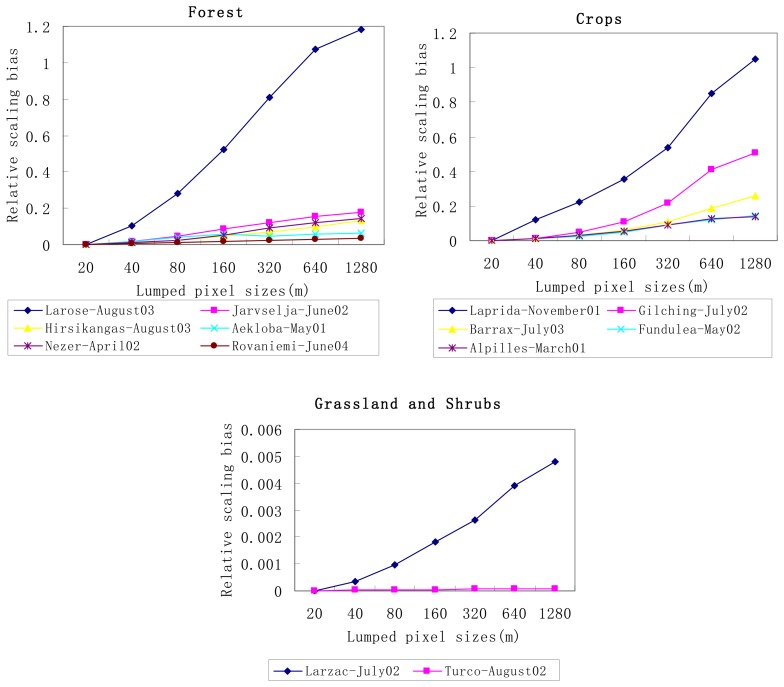
Relative scaling bias of gap probability against pixel size for different landscapes: six forest sites, five crops sites, one grassland site and one shrubs site.

Following the schemes proposed and showed in figure 1, with the VALERI dataset described in table 1, we compute relative scaling bias of gap probability for each site at different spatial scales using Eq. 6. Figure 3 displays the relative scaling bias of gap probability in function of the pixel size for different types of land surfaces, such as forest, cropland, grassland and shrubs.

From this figure, we notice that the relative scaling bias of gap probability increases with decreasing spatial resolution for most of land cover types. Larger relative bias occurs at crops (104%, 50%, 26%, 14%, at pixel size of 1280m, respectively) than pure forest sites (≤ 20% at pixel size of 1280m except for the mixed forest (Larose-August03) which has relative bias of 120% at pixel size of 1280m), grassland and shrubs (≤ 0.5% at pixel size of 1280m), demonstrating that our crops sites are relatively more heterogeneous than forest, grassland and shrubs sites. Previous research conducted by Garrigues et al. (2006b) has gained same conclusion. A large bias occurs over mixed forest site (Larose-August03) due to large variance of LAI with this site, while very small relative biases occur over grassland and shrubs because the variance of LAI over these two sites are small (<0.2) as indicated in table 1.

As a result, a large uncertainty (bias) is introduced in estimate of the gap probability from low spatial resolution data such as NOAA-AVHRR or MODIS over large heterogeneous sites if the scaling effects are not considered.

### 4.3. “Clumping index” Ĉ for VALERI sites

**Figure 4.**
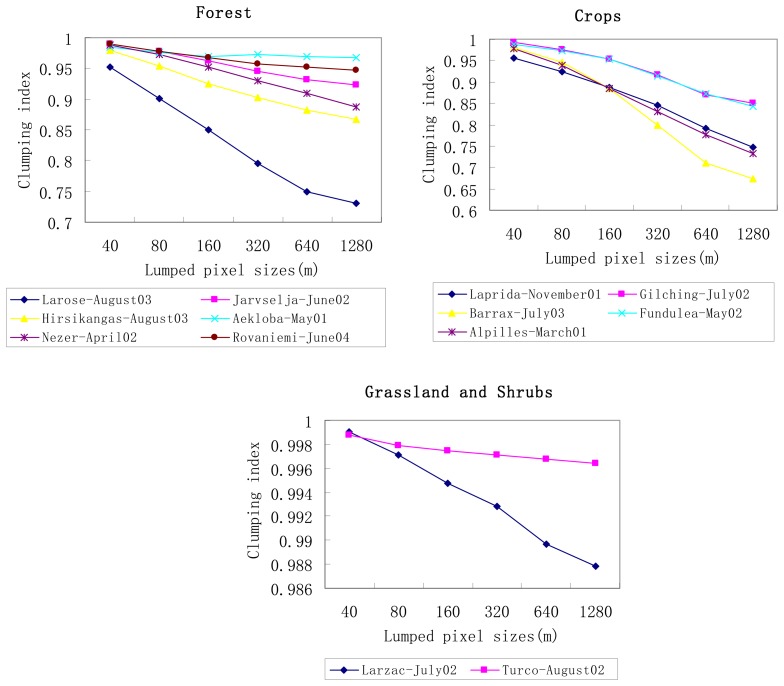
same as figure 3, but with the mean value of clumping index.

Letting Eq. 8 equal to Eq. 2, with VALERI dataset, “clumping index” Ĉ introduced in Eq.7 can be easily obtained for each site at different spatial scales. Figure 4 shows the mean value of “clumping index” against the pixel size for different types of land surfaces, such as forest, cropland, grassland and shrubs. Since the SPOT-HRV pixel is supposed to be homogeneous at 20m spatial resolution, the corresponding “clumping index” Ĉ at original scale is unity (not displayed in figure 4).

As shown in Figure 4, “clumping index” varies much for different land cover types and different aggregated sizes. It decreases as aggregative levels increase, indicating that pixel becomes more heterogeneous as demonstrated by the analysis of the relative scaling bias of gap probability given above. Particularly a relative large variation of “clumping index” occurs at Larose-August03, very similar to the relative scaling bias of gap probability. In addition, “clumping index” varies slowly in pure forest, grassland and shrubs sites and more significantly in crops and mixed forest in our cases study. The results demonstrate that less scaling effect correction should be performed for forest and grass sites than crops sites, which is in good agreement with the result shown in Figure 3.

As far as sites with the same land cover type are concerned, the magnitude of “clumping index” also varies at different aggregated sizes, and mostly is inversely proportional to the spatial heterogeneity of LAI ( $\sigma_{\text{LAI}}^{2}$). For example, among forest sites, “clumping index” is minimum at Aekloba-May01, then Rovaniemi-June04, Jarvselja-June02, Nezer-April02, Hirsikangas-August03, and maximum is at Larose-August03, whose $\sigma_{\text{LAI}}^{2}$ are 0.671, 0.52, 1.09, 1.11, 1.14, 2.00, respectively.

Therefore “clumping index” redefined by Eq. 8 has the capability of representing and eliminating scaling bias of directional gap probability induced by the heterogeneity of LAI.

## 5. Conclusion

In this study, spatial scaling effect of the gap probability based on Beer's law for different types of land cover is analyzed and corrected for by introducing an extension of the “clumping index”, Ĉ which accounts for the spatial heterogeneity.

Analytical expressions developed in this paper show that:

(1) relative scaling bias is only dependent on the G function and the spatial heterogeneity of LAI, but independent on the LAI value itself, and
(2) extension of “clumping index” Ĉ is directly proportional to the mean value of LAI and inversely proportional to the spatial heterogeneity of LAI for given G function and direction.

With the VALERI dataset, this study shows that relative scaling bias of gap probability increases and “clumping index” value decreases with decreasing spatial resolution for most of land cover types. Large relative biases and large variation of “clumping index” Ĉ are found for most of crops sites and a mixed forest site due to their relative large variance of LAI, while very small biases and small variation of clumping index are found for grassland and shrubs sites.

The parameters introduced in this paper has endowed a new significance to traditional clumping index and provided evidence to the utility of clumping index as an improvement of the estimate of gap probability from LAI. The results exhibit the capability of clumping index for scaling Beer' law and representing spatial heterogeneity, as well as the feasibility of the inversion approach for gap probability from remote sensing data. Meanwhile a simple and feasible method to estimate “clumping index” from remote sensing data is also explored from the above experiment, which will provide a support to global mapping of the vegetation clumping index.

## Figures and Tables

**Table 1.** Detailed information of remote sensing images used in this research. The last two columns represent the mean (m) and the standard deviation (σ) of LAI respectively.

**Site name**	**Land cover type**	**Date**	**Lat.**	**Lon.**	*m_LAI_*	*σ_LAI_*
Aekloba-May01	Palm tree plantation	1/Jun./2001	2.63	99.58	3.54	0.671
Alpilles-March01	Crops	15/Mar./2001	43.81	4.74	0.93	1.15
Barrax-July03	Cropland	3/Jul./2003	39.07	-2.10	0.97	1.41
Fundulea-May02	Crops	9/Jun./2002	44.41	26.59	1.53	1.30
Gilching-July02	Crops and forest	8/Jul./2002	48.08	11.32	5.39	1.79
Hirsikangas-August03	Forest	2/Aug./2003	62.64	27.01	2.55	1.14
Jarvselja-June02	Boreal forest	13/Jul./2002	58.30	27.26	4.20	1.09
Laprida-November01	Grassland	3/Nov./2001	-36.99	-60.55	5.66	2.07
Larose-August03	Mixed forest	18/Sep./2003	45.38	-75.21	5.87	2.00
Larzac-July02	Grassland	12/Jul./2002	43.94	3.12	0.81	0.20
Nezer-April02	Pine forest	21/Apr./2002	44.57	-1.04	2.38	1.11
Rovaniemi-June04	Forest	23/Jul./2004	66.46	25.35	1.25	0.52
Turco-August02	Shrubs	29/Aug./2002	-18.24	-68.19	0.04	0.03
